# The cold-induced switch in direction of chloroplast relocation occurs independently of changes in endogenous phototropin levels

**DOI:** 10.1371/journal.pone.0233302

**Published:** 2020-05-21

**Authors:** Yuta Fujii, Yuka Ogasawara, Yamato Takahashi, Momoko Sakata, Minoru Noguchi, Saori Tamura, Yutaka Kodama

**Affiliations:** 1 Center for Bioscience Research and Education, Utsunomiya University, Tochigi, Japan; 2 United Graduate School of Agricultural Science, Tokyo University of Agriculture and Technology, Tokyo, Japan; 3 Faculty of Agriculture, Utsunomiya University, Tochigi, Japan; 4 Graduate School of Agricultural Science, Utsunomiya University, Tochigi, Japan; Rutgers University/New Brunswick, UNITED STATES

## Abstract

When exposed to fluctuating light intensity, chloroplasts move towards weak light (accumulation response), and away from strong light (avoidance response). In addition, cold treatment (5°C) induces the avoidance response even under weak-light conditions (cold-avoidance response). These three responses are mediated by the phototropin (phot), which is a blue-light photoreceptor and has also been reported to act as a thermosensory protein that perceives temperature variation. Our previous report indicated that cold-induced changes in phot biochemical activity initiate the cold-avoidance response. In this study, we further explored the induction mechanism of the cold-avoidance response in the liverwort *Marchantia polymorpha* and examined the relationship between changes in the amount of phot and the induction of the cold-avoidance response. The switch between the accumulation and avoidance responses occurs at a so-called ‘transitional’ light intensity. Our physiological experiments revealed that a cold-mediated decrease in the transitional light intensity leads to the induction of the cold-avoidance response. While artificial overexpression of phot decreased the transitional light intensity as much as cold treatment did, the amount of endogenous phot was not increased by cold treatment in wild-type *M*. *polymorpha*. Taken together, these findings show that the cold-avoidance response is initiated by a cold-mediated reduction of the transitional light intensity, independent of the amount of endogenous phot. This study provides a clue to understanding the mechanism underlying the switch in direction of chloroplast relocation in response to light and temperature.

## Introduction

Plants sense ambient light and temperature to adapt to an ever-changing environment. In response to the light and temperature changes, the intracellular chloroplast position is precisely controlled. Under weak-light conditions (e.g., under canopy), chloroplasts move towards light to increase light capture for photosynthetic efficiency (accumulation response) [1]. Under strong-light conditions (e.g., under direct sunlight), chloroplasts move away from light to avoid photodamage (avoidance response) [2]. Chloroplast position is also affected by temperature [3], notably cold. Under warm conditions, weak light induces the accumulation response; however, under cold conditions (5°C) the same light level induces chloroplast avoidance. This movement referred to as the cold-avoidance (or cold-positioning) response, has been studied in several plant species, including the moss *Funaria hygrometrica* [3], evergreen ferns (e.g., *Adiantum capillus-veneris*) [4], the liverwort *Marchantia polymorpha* [5] and the flowering plant *Arabidopsis thaliana* [6]. In *M*. *polymorpha*, the cold-avoidance response contributes to the reduction of photoinhibition under cold conditions [6].

Earlier studies showed that the accumulation, avoidance and cold-avoidance responses are all mediated by the blue-light (BL) photoreceptor phototropin (phot) [4,6–9]. Phot has two photosensory light, oxygen, or voltage (LOV) domains (LOV1 and LOV2) at its N-terminal region and a serine/threonine kinase domain at its C-terminal region. Phot perceives BL via its LOV domains and mediates the change of chloroplast position upon photoactivation of the LOV domains and kinase-mediated autophosphorylation [10]. A decrease in ambient temperature prolongs the lifetime of the photoactivated LOV2 domain, resulting in an increase in autophosphorylation [6]. The cold-induced enhancement of the photochemical and biochemical activities of phot (i.e., a prolonged lifetime of photoactivated LOV2 and increased autophosphorylation status) is essential for induction of the cold-avoidance response [6], and further study is required to fully understand this induction.

**Fig 1:**
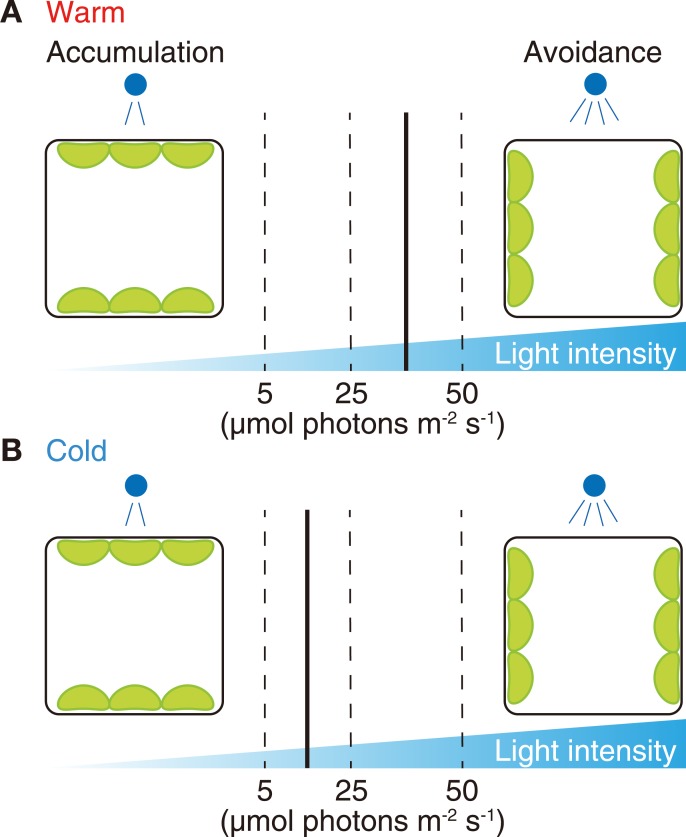
Diagram showing the transitional light intensity for the accumulation and avoidance responses. Solid vertical lines indicate points of the transitional light intensity under warm (A; 22°C) and cold (B; 5°C) conditions. Dashed lines indicate actual intensities of blue light (5, 25 and 50 μmol photons m^-2^ s^-1^) that can induce the accumulation, avoidance and cold-avoidance in *M*. *polymorpha* [6,42].

The switch in chloroplast behavior between the accumulation and avoidance responses occurs at their ‘transitional’ light intensity (Fig 1A). In the cold-avoidance response, because the chloroplast moves away from even weak BL (wBL) under cold conditions [6], it is hypothesized that the cold-avoidance response is induced by a cold-mediated reduction of the transitional light intensity (Fig 1B). However, this hypothesis remains to be tested. Artificial overexpression of phot has been suggested to decrease the transitional light intensity in *A*. *capillus-veneris* and *A*. *thaliana* [11,12]. In addition, expression levels of endogenous phot are affected by both light [7,8,13–16] and temperature [16] conditions. However, to date, there is no evidence that the environment-dependent changes in expression of endogenous phot are involved in the alteration of the transitional light intensity. Based on these findings, we postulate that a variation in the endogenous amount of phot may be involved in the induction of the cold-avoidance response.

The goals of this study were two-fold: first, to determine if a cold-mediated reduction of the transitional light intensity initiates the cold-avoidance response, and second, to determine if increases in phot amounts play a role in the induction of the cold-avoidance response. *M*. *polymorpha* was chosen for this study because it encodes a single copy of phot (Mpphot) that can mediate the accumulation, avoidance, and cold-avoidance responses [6,17].

## Materials and methods

### Plasmid construction

To construct a plasmid for expression of Mpphot-Citrine fusion protein in the Mp*phot* knockout line (Mp*phot*^*KO*^), we used the Gateway cloning system (Invitrogen) and the binary vector pMpGWB306 [18], which includes *Citrine* (a gene for yellow fluorescent protein) for fusion at the 3ʹ-end of the target gene under the control of the cauliflower mosaic virus (CaMV) 35S promoter. The LR reaction (Invitrogen) was performed with a mixture of pMpGWB306 and the donor plasmid pDONR207-MpPHOT [19], and the resulting plasmid pMpGWB306-MpPHOT was used for transformation of the Mp*phot*^*KO*^ line.

### Plant materials and growth conditions

Thalli of *M*. *polymorpha* were asexually cultured on half-strength B5 medium under 75 μmol photons m^-2^ s^-1^ continuous white light (FL40SW, NEC Corporation). Light intensity (photon flux density) was measured by using a LI-250A light meter (LI-COR Biosciences). The Mp*phot*^*KO*^ line was generated and kindly provided by Dr. Takayuki Kohchi (Kyoto University) [17]. In this study, transformation of *M*. *polymorpha* (the Tak-1 strain as wild-type, and the Mp*phot*^*KO*^ line) was performed via the G-AgarTrap method [20,21], which is an *Agrobacterium*-mediated transformation method used for gemmalings. To generate the transformants, we used the binary vector pMpGWB106-MpPHOT [19] for the Mpphot-Citrine overexpression lines (35S::Mpphot-Citrine/WT), pMpGWB102-Citrine [22] for the Citrine expression line (35S::Citrine/WT), and pMpGWB306-MpPHOT for the complementation line of Mpphot (35S::Mpphot-Citrine/Mp*phot*^*KO*^). G2 gemmalings were used throughout.

### Observation of chloroplast positioning

One-day-old Tak-1 gemmalings or 10-day-old sporelings (only for polarized weak BL experiment) were incubated in temperature-controlled incubators (IJ100, Yamato Scientific Co., LTD) and light conditions were adjusted using blue light-emitting diodes (LEDs; OptoSupply Limited) and a LI-250A light meter. To produce polarized weak BL, 25 μmol photons m^-2^ s^-1^ of the blue LED was used as weak BL [5,6], and it was filtered with a polarizing filter (HN38, Polaroid Corp.). Note that the term “weak BL” in the present study denotes BL that induces the accumulation response in *M*. *polymorpha* at 22°C. Chlorophyll fluorescence images were captured using a fluorescence microscope MZ16F (Leica Microsystems) equipped with a DP73 digital camera (Olympus) and quantification of chloroplast positioning was performed using the P/A ratio method with ImageJ software, as previously described [5]. Briefly, fluorescence intensity was measured at 30 points (0.625 μm each, equivalent to 1 pixel) along the anticlinal cell walls (A) and 30 points (39.1 μm^2^ each, equivalent to 10 × 10 pixels) along the periclinal cell walls (P). This procedure was repeated five times, and then the P/A ratio was calculated as a mean of the five P/A values. The standard deviation was calculated using Excel software (Microsoft).

### Immunoblot analysis

Immunoblot analysis was performed as previously described [6]. Briefly, four-day-old gemmalings were collected in liquid nitrogen and kept at −80°C until use. The frozen plants were homogenized in SDS buffer (2% SDS, 10% glycerol, 5% mercaptoethanol, and 0.25 M Tris-HCl pH6.8) or Nonidet buffer (0.5% Nonidet P-40, 0.5M EDTA, 0.15 M NaCl, and 0.01 M Tris-HCl pH7.5) using a mortar and pestle and then boiled at 95°C for 5 min. The samples were centrifuged at 14,000 g for 10 min, and the supernatants used. Total protein amount was quantified by Bradford-XL assay (APRO Science). Fifteen μg of protein was subjected to SDS-PAGE with 8% polyacrylamide gels (acrylamide: *N*,*Nʹ*-methylenebisacrylamide = 37.5:1) and transferred to a polyvinylidene difluoride (PVDF) membrane [6]. For gel-mobility shift assay to detect phosphorylated Mpphot, modified 8% polyacrylamide gels (acrylamide: *N*,*Nʹ*-methylenebisacrylamide = 29.9:0.1) were used [6]. Phosphatase treatment to dephosphorylate Mpphot was previously described [6]. Mpphot proteins were detected with rabbit antibody raised against the custom-made synthetic peptide (C+VDERAPPSKGSAKE) (Eurofins Genomics) and horseradish peroxidase-conjugated anti-rabbit IgG (Thermo Scientific). Both antibodies were used at dilution of 1:2000. For histone detection, anti-Histone H3 antibody (Abcam) at dilution of 1:5000 and horseradish peroxide-conjugated anti-rabbit IgG (Thermo Scientific) at dilution of 1:2000 were used. Chemiluminescence signal was detected using ECL-Select (GE Healthcare) and Light Capture (ATTO). To visualize Rubisco large subunit (RBCL), fifteen μg protein was subjected to SDS-PAGE with 8% polyacrylamide gels and stained with conventional Coomassie brilliant blue (CBB) method.

## Results

### Chloroplasts avoid weak blue light (wBL) under cold conditions

Using temperature-regulated microscopy with a microbeam irradiation system, we previously reported that under cold conditions (5°C), chloroplasts move away from wBL of an intensity that induced the accumulation response at 22°C [6]. The results suggest that the cold-avoidance response is similar to the avoidance response induced by strong light, and that there is a transitional light intensity that serves as an inflection point between the accumulation and cold-avoidance responses. However, physiological evidence of wBL avoidance under cold conditions was limited to microbeam analysis [6]. Here, to further analyze whether chloroplasts move away from wBL under cold conditions, we performed experiments with light direction changes and polarized light, similar to the previous studies with *A*. *capillus-veneris* [23,24].

**Fig 2:**
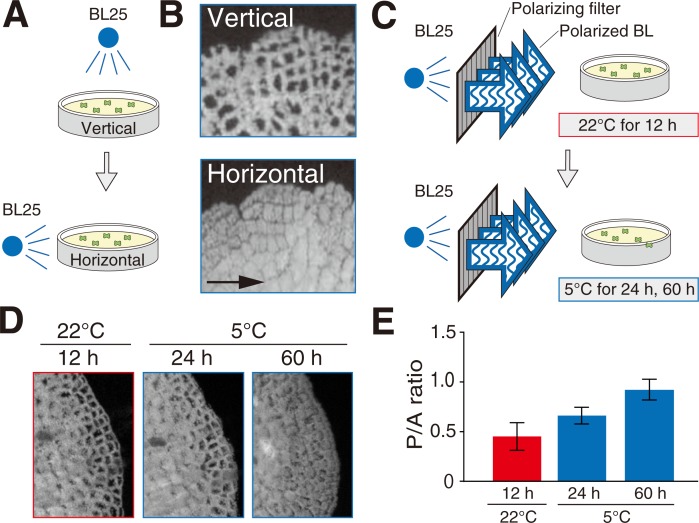
Chloroplasts avoid weak blue light under cold conditions. (A) Diagram of the experimental conditions used to induce the cold-avoidance response with vertical or horizontal irradiation of blue light. After incubation under 25 μmol photons m^-2^ s^-1^ of blue light (BL25) with vertical irradiation at 5°C for 24 h, the WT gemmalings were irradiated by horizontal BL25. (B) Chlorophyll fluorescence images after irradiation of vertical or horizontal BL25 as shown in (A). The arrow indicates the light direction. (C) Diagram of the experimental conditions used to induce the accumulation response at 22°C and cold-avoidance response at 5°C with horizontal irradiation of vertically vibrating polarized weak BL. Ten-day-old WT sporelings were irradiated by the polarized weak BL (BL25) at 22°C for 12 h. The cells were transferred to 5°C, followed by incubation for 24 h and 60 h. (D) Chlorophyll fluorescence images after irradiation of the polarized weak BL as shown in (C). (E) P/A ratios calculated from chlorophyll fluorescence images shown in (D). Error bars indicate standard deviations.

In *M*. *polymorpha*, both cold and BL were required to elicit the cold-avoidance response [6]. Building on that result, to explore whether the cold-avoidance response is due to avoidance of wBL under cold conditions, we tested the effects of changing light direction. First, to induce the cold-avoidance response, wild-type (WT) cells were incubated under 25 μmol photons m^-2^ s^-1^ of wBL (BL25) with vertical irradiation at 5°C (Fig 2A). In WT cells, chloroplasts were positioned along the anticlinal cell walls as the cold-avoidance response (Fig 2B). When the light direction was changed from vertical to horizontal (Fig 2A), the chloroplasts originally along the anticlinal cell walls moved to the periclinal cell walls (Fig 2B). The cold-avoidance response depended on the direction of BL25, and chloroplasts avoided BL25 at 5°C (Fig 2A and 2B). To further validate the results, we employed polarized wBL, because horizontal irradiation with the vertically vibrating polarized wBL induces chloroplast localization along the anticlinal wall via the accumulation response in *A*. *capillus-veneris* [24]. Chloroplast position in *M*. *polymorpha* was quantified by a P/A ratio method, developed in earlier studies [4,5]. The P/A ratio is an arbitrary unit calculated by dividing the chlorophyll fluorescence intensity along the periclinal cell wall by that along the anticlinal cell wall, with a higher value indicating periclinal positioning of chloroplasts and vice versa. When 10-day-old sporeling cells of *M*. *polymorpha* were horizontally irradiated with vertically vibrating polarized wBL for 12 h at 22°C (Fig 2C), chloroplasts localized along the anticlinal wall (Fig 2D and 2E), representing an accumulation response. When the cells were transferred to 5°C under the same polarized wBL condition (Fig 2C), chloroplasts relocated from the anticlinal wall to the periclinal wall and the P/A ratio was increased (Fig 2D and 2E), indicating that chloroplasts avoided the polarized wBL at 5°C. These results confirm that the cold-avoidance response is due to avoidance of wBL under cold conditions, which is consistent with the microbeam analysis in our previous study [6]. The results suggest that there is the transitional light intensity between the accumulation and cold-avoidance responses, like that between the accumulation and avoidance responses (Fig 1).

### Cold treatment decreases the transitional light intensity between the accumulation and avoidance responses

**Fig 3:**
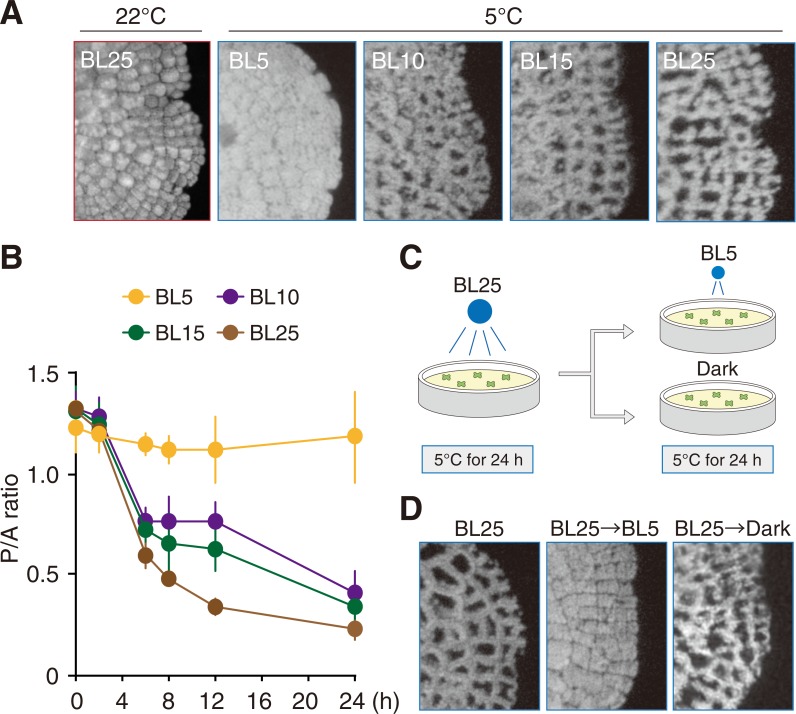
Cold-induced reduction of the transitional light intensity. (A) Chloroplast positioning in WT gemmalings incubated at 5°C for 12 h with 5, 10, 15, and 25 μmol photons m^-2^ s^-1^ of blue light (BL5, BL10, BL15, and BL25, respectively). After pre-incubation of 1-day-old WT gemmalings under BL25 at 22°C for 1 h to induce the accumulation response, the cells were transferred to the indicated BL conditions at 5°C. (B) The P/A ratios, calculated from chlorophyll fluorescence images after incubation for 0, 2, 6, 8, 12, and 24 h. Error bars indicate standard deviations. (C) Diagram of experimental conditions used to demonstrate the cold-induced reduction of the transitional intensity. After pre-incubation under BL25 at 5°C for 24 h, WT gemmalings were transferred to BL5 or dark and incubated at 5°C for 24 h. (D) Representative chlorophyll fluorescence images of WT gemmalings after pre-incubation (BL25) and incubation under BL5 (from BL25 to BL5) or dark (from BL25 to Dark) as shown in (C).

If there is the transitional light intensity between the accumulation and cold-avoidance responses, the accumulation response should be induced under much weaker BL in cold conditions. To investigate whether the accumulation response is induced at 5°C, WT cells were incubated under different intensities of wBL (5, 10, 15 and 25 μmol photons m^-2^ s^-1^; BL5, BL10, BL15 and BL25, respectively) (Fig 3A and 3B). In WT cells, the cold-avoidance response was observed at 5°C under BL10, BL15 and BL25 (Fig 3A), and at these higher BL intensity levels, we observed a decrease in P/A ratios (Fig 3B). However, the cold-avoidance response was not observed at 5°C under BL5 (Fig 3A) and the P/A ratio was unchanged during the incubation of these samples (Fig 3B). These results suggest that BL5 induces the accumulation response at 5°C. However, because similar chloroplast positioning was observed under dark conditions at 5°C [6], it is also possible that the cells were unable to respond to the extremely weak intensity of BL5. To clarify whether the accumulation response is induced under BL5 or not, an additional test was carried out. Following the induction of the cold-avoidance response at 5°C for 24 h under BL25, the light intensity was changed to BL5 or dark conditions (Fig 3C). After incubation for 24 h, BL5 conditions induced the accumulation response at 5°C, whereas under dark conditions there was no change in chloroplast position (Fig 3D). These results indicate that the accumulation response is induced under BL5 at 5°C. Taken together, they indicate that the transitional light intensity is modulated by the temperature change (Fig 1).

### Artificial overexpression of Mpphot in *M*. *polymorpha* induces the avoidance response under wBL without cold treatment

**Fig 4:**
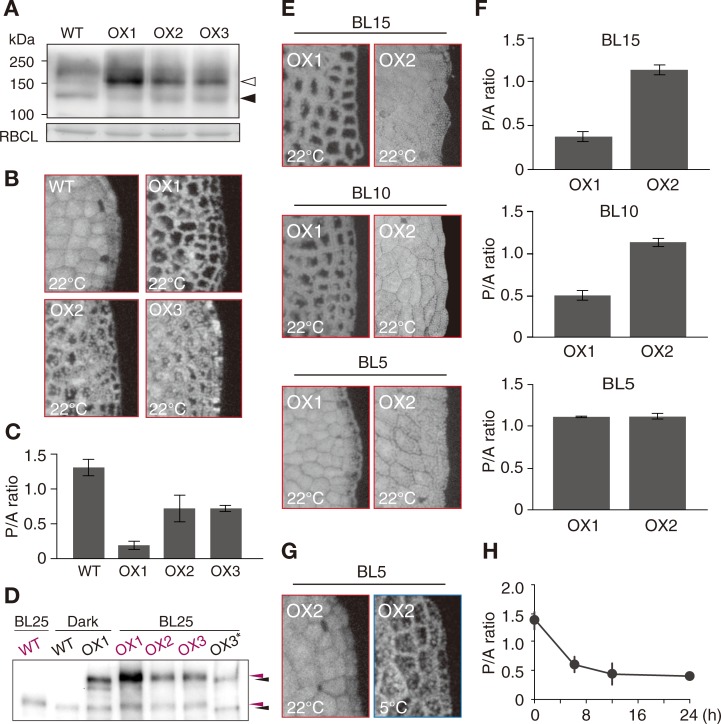
Overexpression of Mpphot-Citrine reduces the transitional light intensity. (A) Immunoblot analysis of WT and Mpphot-Citrine overexpression lines (35S::Mpphot-Citrine/WT: OX1, OX2, and OX3) with anti-Mpphot antibody. White and black arrowheads indicate Mpphot-Citrine and Mpphot, respectively. Rubisco large subunit (RBCL) was shown as a loading control. (B) Representative images of chlorophyll fluorescence in WT and Mpphot-Citrine overexpression lines after the incubation under BL25 at 22°C for 12 h. (C) P/A ratios calculated from the chlorophyll fluorescence images shown in (B). (D) Gel-mobility shift assay to detect BL-dependent phosphorylated forms of native Mpphot and Mpphot-Citrine in WT and Mpphot-Citrine overexpression lines (OX1, OX2, and OX3) at 22°C. Four-day-old gemmae were incubated under dark condition for 72 h or BL25 condition for 12 h. Immunoblot analysis was performed with anti-Mpphot antibody. Magenta arrowheads indicate the band positions of BL-dependent phosphorylated forms (WT, OX1, OX2, and OX3) of Mpphot-Citrine (upper) and native Mpphot (lower). Black arrowheads indicate the band positions of dark-adapted (WT and OX1) and dephosphorylated (OX3*) forms of Mpphot-Citrine (upper) and native Mpphot (lower). The asterisk indicates the dephosphorylated OX3 sample by phosphatase treatment (10 units of FastAP thermosensitive alkaline phosphatase, Thermo Fisher Scientific) [6]. (E) Representative images of chlorophyll fluorescence in WT and Mpphot-Citrine overexpression lines (OX1 and OX2) after incubation under BL15, BL10 or BL5 at 22°C for 12 h. (F) P/A ratios calculated from the chlorophyll fluorescence images shown in (E). (G) Confirmation of the reduction of transitional light intensity in OX2 under cold conditions. In the 1-day-old gemmalings from OX2, the accumulation response was induced under BL5 at 22°C (left panel). The cold-avoidance response was induced under BL5 at 5°C for 24 h (right panel). (H) Time-course of the cold-avoidance response induced in OX2 under BL5 at 5°C. The P/A ratios were calculated from the chlorophyll fluorescence images after incubation for 0, 6, 12, and 24 h.

We next considered the possibility that increases in endogenous phot expression may play a role in the decrease in transitional light intensity, as previous studies using *A*. *capillus-veneris* and *A*. *thaliana* suggest that a reduction in the transitional light intensity can be the result of artificial overexpression of phot [11,12]. In *A*. *capillus-veneris* at 25°C, transient overexpression of the Ac*PHOT2-GFP* gene induced the avoidance response under wBL [11]. Similarly, the avoidance response was observed under wBL in transgenic *A*. *thaliana* overexpressing the At*PHOT2-GFP* gene at 23°C [12]. Consistent with these previous studies, we observed that artificial overexpression of Mpphot fused to the yellow fluorescent protein Citrine (Mpphot-Citrine) in WT cells (35S::Mpphot-Citrine/WT) induced the avoidance response under BL25 at 22°C (without cold treatment), and degree of the avoidance response was dependent on Mpphot-Citrine expression levels (Fig 4A–4C). Notably, in the overexpression (OX) lines incubated under BL25 for 12 h, both native Mpphot and Mpphot-Citrine were phosphorylated (Fig 4D). When OX1 (a higher overexpression line) and OX2 (a lower overexpression line) were incubated under BL15 and BL10 at 22°C, the avoidance response was induced in the OX1 line, whereas the accumulation response was induced in the OX2 line (Fig 4E and 4F). By contrast, under BL5 at 22°C, the accumulation response was induced in both OX1 and OX2 lines (Fig 4E and 4F). When the OX2 line was incubated under BL5 at 5°C, the cold-avoidance response was induced (Fig 4G and 4H), indicating that the transitional light intensity is reduced by overexpression of Mpphot-Citrine. Therefore, it is conceivable that increases in the endogenous amount of Mpphot might play a role in the induction of the cold-avoidance response.

### Alteration of endogenous Mpphot is not required for the induction of the cold-avoidance response

**Fig 5:**
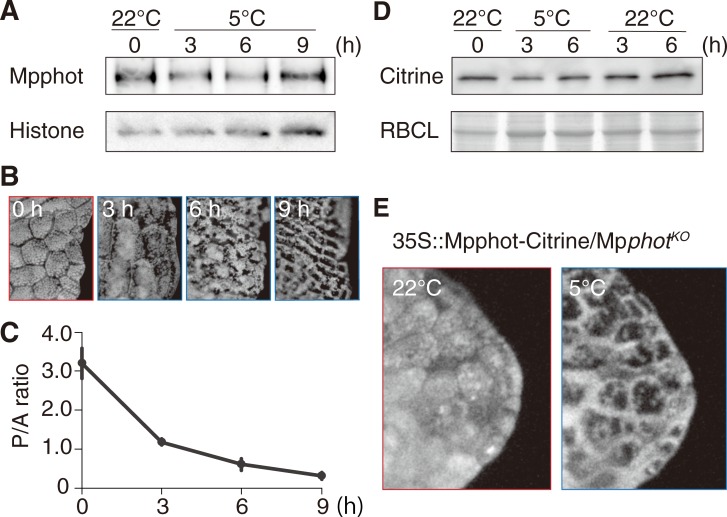
Increased Mpphot expression is not required for the induction of the cold-avoidance response. (A) Immunoblot analysis of endogenous Mpphot in 4-day-old WT gemmalings (white light for 3 days and BL25 for 24 h at 22°C), incubated under BL25 for 0 h (22°C), and 3 h, 6 h and 9 h at 5°C. Histone H3 protein is shown as a loading control. (B) Representative chlorophyll fluorescence images of WT gemmalings incubated under the same light and temperature conditions as shown in (A). (C) P/A ratios calculated from chlorophyll fluorescence images after incubation for 0 h (22°C), and 3 h, 6 h and 9 h at 5°C. Error bars indicate standard deviations. (D) Immunoblot analysis of activity of CaMV35S promoter in *M*. *polymorpha* under cold conditions. A transformant expressing Citrine under the control of CaMV35S promoter (35S::Citrine/WT) was used. Coomassie Brilliant Blue (CBB)-stained Rubisco large subunit (RBCL) was used as a loading control. (E) Chlorophyll fluorescence images showing the cold-avoidance response in complementation lines (35S::Mpphot-Citrine/Mp*phot*^*KO*^). After incubation under BL25 at 22°C, the gemmalings were transferred to 5°C under the same light conditions.

To examine the role of Mpphot expression in the induction of the cold-avoidance response, the endogenous amount of Mpphot was determined using immunoblot analysis. After gemmalings were transferred from white light to BL25 at 22°C, the endogenous amount of Mpphot was increased at 6 h and the increased amount was maintained until 24 h (S1 Fig). To avoid the effect of the light condition change (i.e., from white light to BL25), the gemmalings were pre-incubated under BL25 at 22°C for 24 h. When the temperature was changed from 22°C to 5°C, the endogenous amount of Mpphot was not drastically changed until 9 h though a slight but statistically significant decrease was observed at 6 h (Fig 5A and S2 Fig). Note that this experimental set up was not able to distinguish between phosphorylated and dephosphorylated forms of Mpphot. When we incubated the gemmalings under the same light and temperature conditions as shown in Fig 5A, the cold-avoidance response was induced (Fig 5B), and the P/A ratio was decreased (Fig 5C). Unexpectedly the pre-incubation under BL25 at 22°C for 24 h increased the P/A ratio compared to the white light condition at 22°C (see 0 h in Fig 5C and 0 h in Fig 3B). Overall, we concluded that the cold-avoidance response in *M*. *polymorpha* could be induced without any clear change in Mpphot abundance during cold treatment. To validate this result, we examined the complementation lines of Mpphot-Citrine under the control of the CaMV35S promoter in the genetic background of Mp*phot*^*KO*^ (35S::Mpphot-Citrine/Mp*phot*^*KO*^), as CaMV35S promoter activity is reported to be suppressed by cold treatment in tobacco plants (i.e., no enhancement of the CaMV35S promoter activity under cold conditions) [25]. Before the experiments with 35S::Mpphot-Citrine/Mp*phot*^*KO*^, we produced transgenic *M*. *polymorpha* expressing Citrine under the control of the CaMV35S promoter (35S::Citrine/WT) to confirm that there was no enhancement of the CaMV35S promoter activity in *M*. *polymorpha* under cold conditions. In the 35S::Citrine/WT, Citrine expression was unchanged by 5°C treatment (Fig 5D and S3 Fig). The results indicate no enhancement of the CaMV35S promoter activity in *M*. *polymorpha* under cold conditions. In the complementation lines (35S::Mpphot-Citrine/Mp*phot*^*KO*^), the cold-avoidance response was clearly observed (Fig 5E), confirming that the induction of the cold-avoidance response does not require increase of the Mpphot amount. Given that cold treatment increased the autophosphorylation status of phot [6,26], we conclude that an increase of phot autophosphorylation decreases the transitional light intensity without altering the endogenous amount of Mpphot.

## Discussion

### The cold-avoidance response is a phenomenon wherein chloroplasts move away from wBL at low temperature

Cold-induced chloroplast relocation from the periclinal wall to anticlinal wall under weak-light conditions have been observed over the past century [3], and this relocation response was originally called “cold positioning” [3,4]. In our previous study, we performed microbeam experiment to understand chloroplast behavior in response to wBL under cold conditions. When *M*. *polymorpha* cell temperature was precisely controlled at 5°C using temperature-regulated microscopy, chloroplasts clearly moved away from a wBL microbeam [6]. Based on this behavior, we renamed cold positioning as the cold-avoidance response [6]. In the present study, we reconfirmed the behavior that chloroplasts avoid wBL under cold conditions, this time using wBL direction changes and polarized wBL (Fig 2). In *A*. *capillus-veneris*, the red-light- or BL-induced accumulation response was previously analyzed using similar light direction changes and polarized light [23,24]. Based on the previous studies in *A*. *capillus-veneris* and the present study in *M*. *polymorpha*, these experimental methods using light direction change and polarized light are suitable to observe both the cold-avoidance and the accumulation responses.

Notably, vertically vibrating polarized wBL seems to stimulate only the phot localized on or near the anticlinal plasma membrane [24,27]. Although Mpphot localizes to multiple cellular compartments such as the plasma membrane, cytosol, and chloroplast periphery in *M*. *polymorpha* [28], the plasma-membrane-localized pool of Mpphot might mediate the cold-avoidance response via sensing both wBL and low temperature. Alternatively, given that a relationship between the cold-avoidance response and localization at the chloroplast periphery was also suggested [28], it is possible that Mpphot populations localized at both the plasma membrane and chloroplast periphery cooperatively mediate the cold-avoidance response. Further study is needed to conclude subcellular localization(s) of Mpphot responsible for the cold-avoidance response.

### The transitional light intensity between the accumulation and avoidance responses can be changed

Light-induced chloroplast movement is widely observed in photosynthetic organisms from algae to land plants [3]. The transitional light intensity between the accumulation and avoidance responses differs among various plant species [29]. Furthermore, the transitional light intensity can be reduced in response to low temperature, inducing the cold-avoidance response [4–6]. Because the cold-avoidance response moderates photodamage in *M*. *polymorpha* [6], the plasticity of the transitional light intensity is vital for plants exposed to low temperatures. In the present study, we analyzed the light fluence dependency of the cold-avoidance response. Under cold conditions, chloroplasts moved in a fluence rate-dependent manner (Fig 3A and 3B), as occurs during the avoidance response at standard growth temperatures [30]. This result suggests that mechanism of the phot-mediated light responsiveness during the cold-avoidance response is identical to that during the avoidance response, even though their transitional light intensities are different (Fig 1).

In *A*. *thaliana*, two types of phot that mediate light-induced chloroplast movement have been described: Atphot1 and Atphot2. Atphot1 specifically regulates the accumulation response, while Atphot2 regulates both the accumulation and avoidance responses [7–9]. Previous studies revealed that the transitional light intensity in the At*phot1* mutant is similar to that in WT plants, suggesting that Atphot2 mainly controls the transitional light intensity in *A*. *thaliana* [31,32]. In addition, the transitional light intensity between the accumulation and avoidance responses is lowered by an engineered Atphot2 with a long-lifetime of the photoactivated state [33]. Moreover, alteration of the transitional light intensity between the accumulation and avoidance responses was observed in *A*. *thaliana* plants mutated in a red-light photoreceptor (phytochrome B) and the regulation factors involved in the accumulation response (JAC1 and RPT2/NCH1) [31,34–36]. As mentioned above, given the similarity of the phot-mediated light responsiveness during the cold-avoidance response and the classical avoidance response, phytochrome B, JAC1 and RPT2/NCH1 may similarly affect the transitional light intensity between the accumulation and cold-avoidance responses. Further analysis of the transitional light intensity in various plant species and genetic mutants may lead to a better understanding of the transition mechanism of chloroplast relocation movements.

### The amount of photoactivated phototropin influences the transitional light intensity

Intracellular phot proteins are more frequently activated under strong light than weak-light conditions. Weak-light irradiation at lower temperatures, prolongs the lifetime of photoactivated phot [6], and like the effect of strong-light irradiation at a standard growth temperature, results in the increase of photoactivated phot population. Therefore, we concluded that the intracellular amount of photoactivated phot determines the transition between the accumulation and avoidance responses [6]. Furthermore, considering the effect of Mpphot overexpression on the transitional light intensity (Fig 4), we hypothesize that the transition mechanism is based on the absolute amount of photoactivated phot in the cell rather than the ratio of active/inactive phot.

In the cells of the OX lines, the spatial density of Mpphot is believed to be increased, compared with that in WT, because cell size of the OX lines does not appear to be larger than to that of WT (Fig 4B). The increased density should increase the amount of photoactivated Mpphot in the OX lines under the same photon flux density. How an increase in photoactivated phot proteins triggers the switch from the accumulation to avoidance responses remains unknown. Because phot autophosphorylation is also increased under strong-light/warm or weak-light/cold conditions [6,26,37], we speculate that to induce the avoidance response, an increase of photoactivated phot amount may change the phot phosphorylation status, possibly via intermolecular phosphorylation activity [38,39]. Indeed, the absolute amount of phosphorylated Mpphot (i.e., phosphorylated native Mpphot plus phosphorylated Mpphot-Citrine) was increased in the OX lines, in which the amount of photoactivated Mpphot (i.e., photoactivated native Mpphot plus photoactivated Mpphot-Citrine) also seems to be increased (Fig 4D). On the other hand, the phosphorylation level of native Mpphot in the OX lines was similar to that in WT line, suggesting that the ratio of active/inactive Mpphot is comparable between the OX and WT lines (Fig 4D). The results support our hypothesis on the transition mechanism. Interestingly, the recruitment of monomeric phot to sterol-rich microdomains at the plasma membrane to form phosphorylated dimers is involved in phot autophosphorylation [40]. Further autophosphorylation analysis regarding the intracellular spatial density of photoactivated phot may clarify the switching mechanism between the accumulation and avoidance responses.

### Alteration of phot expression is not required for induction of the cold-avoidance response in *M*. *polymorpha*

Many previous studies revealed light-induced changes in expression of *phot* mRNA and proteins in plant species including *A*. *thaliana*, *Oryza sativa* and *Zea mays* [7,8,13–16]. Although not all data in the previous studies were obtained from leaf experiments, it is possible that the light-induced changes in phot expression affect the transitional light intensity between the accumulation and avoidance responses. However, the accumulation and avoidance responses are initiated within several minutes after the onset of light irradiation [8], although light-induced changes in expression of *phot* mRNA and proteins could be detected after several hours [7,8,13–16]. These previous data suggest that light-induced changes in expression level of *phot* mRNA and proteins are not involved in the initiation of the accumulation and avoidance responses.

In *A*. *thaliana*, the endogenous amounts of Atphot1 and Atphot2 change in response to temperature; lower temperature decreases Atphot1 expression and increases Atphot2 expression [16]. In the present study, we showed that the cold-avoidance response can be induced regardless of the increase in the endogenous Mpphot amount (Fig 5A–5C) and even without its promoter (Fig 5D and 5E), indicating that transcriptional and translational regulation of Mpphot is not required for the cold-avoidance response in *M*. *polymorpha*. Mpphot is similar to Atphot2, which regulates both the accumulation and avoidance responses [17]. In *A*. *thaliana*, Atphot2 also mediates the cold-avoidance response [6]. Similarly to Mpphot, Atphot2 levels in *A*. *thaliana* was reported to be unchanged at a temperature range from 4°C to 23°C [16]. Notably, cold treatment had a more significant effect on the decrease of Atphot1 levels than the increase of Atphot2 levels [16], implying that the expression of Atphot1 might be more sensitive to cold than that of Atphot2. Previous studies indicated that Atphot1 and Atphot2 have the high and low activities, respectively, to induce the accumulation response in *A*. *thaliana* [31,32]. In this context, the cold-induced decrease of Atphot1 levels may contribute rapid physiological change from the accumulation response to cold-avoidance response in *A*. *thaliana*. The gene duplication of *phot* [41] and functional differentiation between phot1 and phot2 may have enabled land plants to have variable transitional light intensities and chloroplast movements, allowing species to expand their range of habitat.

## Supporting information

S1 Fig Effect of the light condition change on the endogenous Mpphot expression. Immunoblot analysis of endogenous Mpphot amount in WT gemmaling, incubated under BL25 at 22°C after culture under the white light condition (75 μmol photons m^-2^ s^-1^) at 22°C for 3 days. The black arrowhead and asterisk indicate Mpphot and non-specific signal, respectively. Histone H3 protein is shown as a loading control.
(PDF)

S2 Fig Quantification of endogenous Mpphot in *M*. *polymorpha* under cold conditions. Quantitative data for the endogenous Mpphot amount in *M*. *polymorpha* under cold conditions are shown (representative images in Fig 5A). Signal intensities of Citrine (immunoblotting) and RBCL (CBB staining) were measured using ImageJ, and the Citrine intensity was normalized to the RBCL intensity. An averaged intensity of 0 h (22°C) was set to 1, and relative intensities of 3 h, 6 h and 9 h at 5°C were determined. Data represent the mean ± standard deviation of three independent experiments (see S1 Raw images for Fig 5A). Statistical analysis was performed by one-way ANOVA followed by Dunnett’s test. NS indicates no significant difference at P > 0.05, and an asterisk indicates significant difference at P < 0.05.
(PDF)

S3 Fig Quantification of CaMV35S promoter activity in *M*. *polymorpha* under cold conditions. Quantitative data of CaMV35S promoter activity in *M*. *polymorpha* under cold conditions are shown (representative images in Fig 5D). Signal intensities of Citrine (immunoblotting) and RBCL (CBB staining) were measured using ImageJ, and the Citrine intensity was normalized to the RBCL intensity. An averaged intensity of 0 h (22°C) was set to 1, and relative intensities of 3 h (5°C), 6 h (5°C), 3 h (22°C) and 6 h (22°C) were determined. Data represent the mean ± standard deviation of three independent experiments (see S1 Raw images for Fig 5D). Statistical analysis was performed by one-way ANOVA followed by Dunnett’s test, and there was no statistically significant difference.
(PDF)

S1 Raw images (PDF)
