# Supplementary material for: The cold-induced switch in direction of chloroplast relocation occurs independently of changes in endogenous phototropin levels
Source: PLoS One. 2020 May 21;15(5):e0233302. doi: 10.1371/journal.pone.0233302 (PMC7241815; doi:10.1371/journal.pone.0233302)
Supplement: S1 Fig — Immunoblot analysis of endogenous Mpphot amount in WT gemmaling, incubated under BL25 at 22°C after culture under the white light condition (75 μmol photons m-2 s-1) at 22°C for 3 days. The black arrowhead and asterisk indicate Mpphot and non-specific signal, respectively. Histone H3 protein is shown as a loading control. (PDF) [file pone.0233302.s001.pdf]

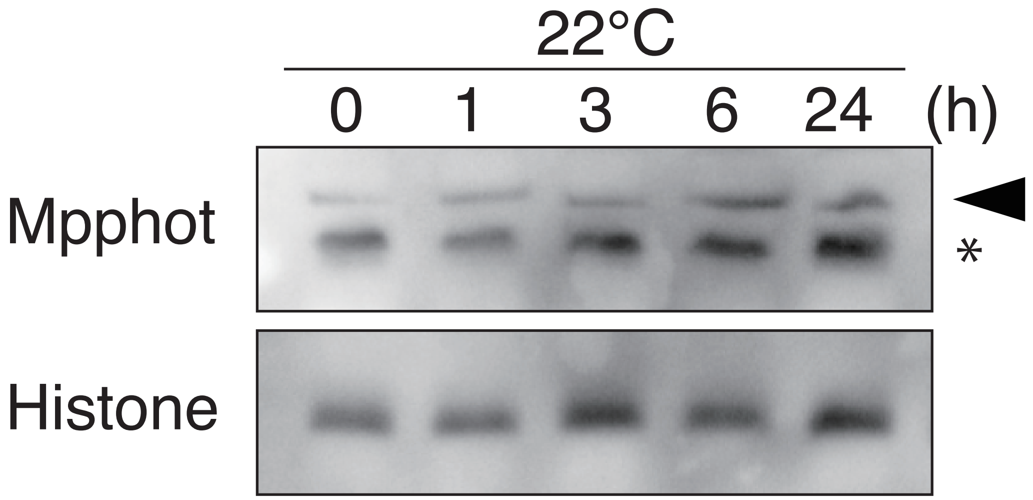

S Fig 1. Effect of the light condition change on the endogenous Mpphot expression. Immunoblot analysis of endogenous Mpphot amount in WT gemmaling, incubated under BL25 at 22° C after culture under the white light condition (75 µmol photons m^{-2} s^{-1}) at 22° C for 3 days. The black arrowhead and asterisk indicate Mpphot and non-specific signal, respectively. Histone H3 protein is shown as a loading control.
