# Supplementary material for: The cold-induced switch in direction of chloroplast relocation occurs independently of changes in endogenous phototropin levels
Source: PLoS One. 2020 May 21;15(5):e0233302. doi: 10.1371/journal.pone.0233302 (PMC7241815; doi:10.1371/journal.pone.0233302)
Supplement: S3 Fig — Quantitative data of CaMV35S promoter activity in M. polymorpha under cold conditions are shown (representative images in Fig 5D). Signal intensities of Citrine (immunoblotting) and RBCL (CBB staining) were measured using ImageJ, and the Citrine intensity was normalized to the RBCL intensity. An averaged intensity of 0 h (22°C) was set to 1, and relative intensities of 3 h (5°C), 6 h (5°C), 3 h (22°C) and 6 h (22°C) were determined. Data represent the mean ± standard deviation of three independent experiments (see S1 Raw images for Fig 5D). Statistical analysis was performed by one-way ANOVA followed by Dunnett’s test, and there was no statistically significant difference. (PDF) [file pone.0233302.s003.pdf]

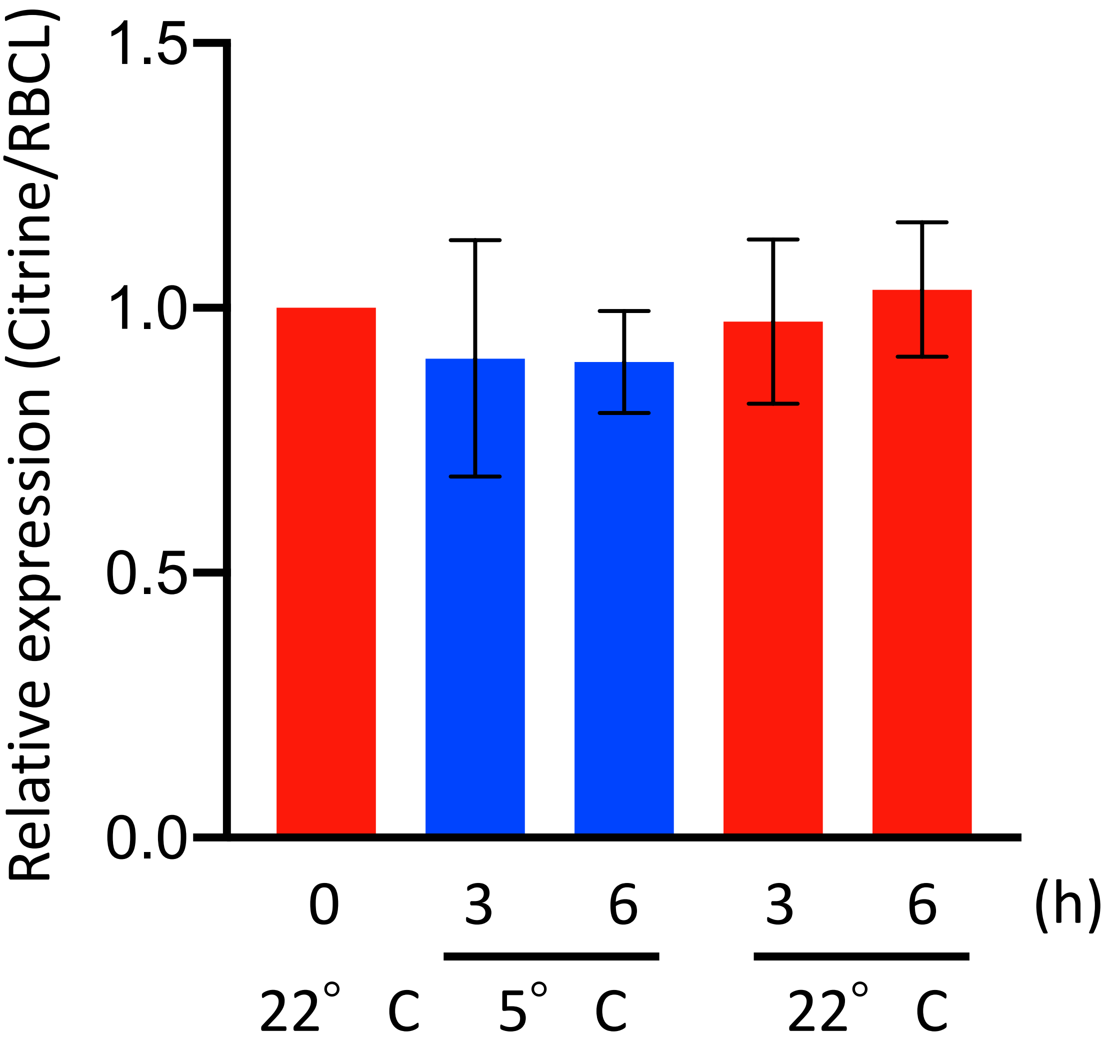

S Fig 3. Quantification of CaMV35S promoter activity in *M. polymorpha* under cold conditions. Quantitative data of CaMV35S promoter activity in *M. polymorpha* under cold conditions are shown (representative images in Fig 5D). Signal intensities of Citrine (immunoblotting) and RBCL (CBB staining) were measured using ImageJ, and the Citrine intensity was normalized to the RBCL intensity. An averaged intensity of 0 h (22° C) was set to 1, and relative intensities of 3 h (5° C), 6 h (5° C), 3 h (22° C) and 6 h (22° C) were determined. Data represent the mean ± standard deviation of three independent experiments (see S1_raw_images for Fig 5D). Statistical analysis was performed by one-way ANOVA followed by Dunnett’s test, and there was no statistically significant difference.
