# Supplementary material for: The cold-induced switch in direction of chloroplast relocation occurs independently of changes in endogenous phototropin levels
Source: PLoS One. 2020 May 21;15(5):e0233302. doi: 10.1371/journal.pone.0233302 (PMC7241815; doi:10.1371/journal.pone.0233302)

Figure 4A

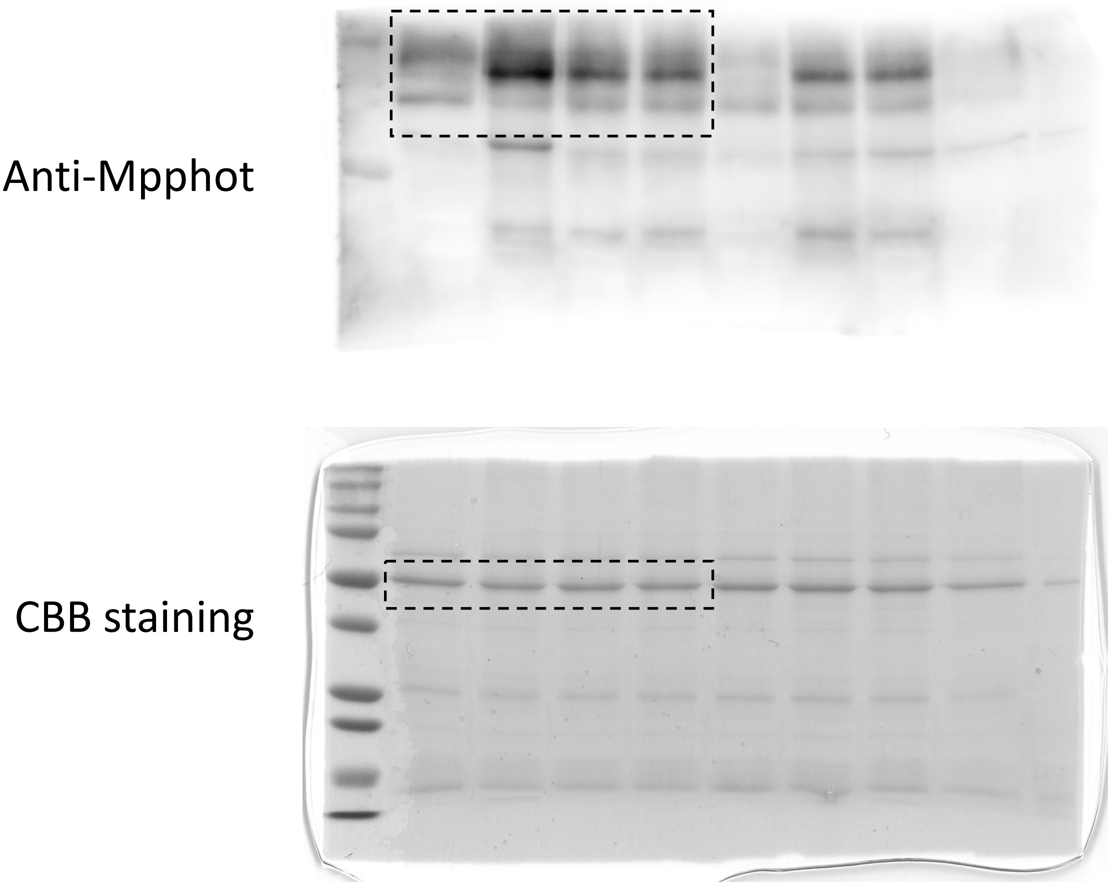

Figure 4D

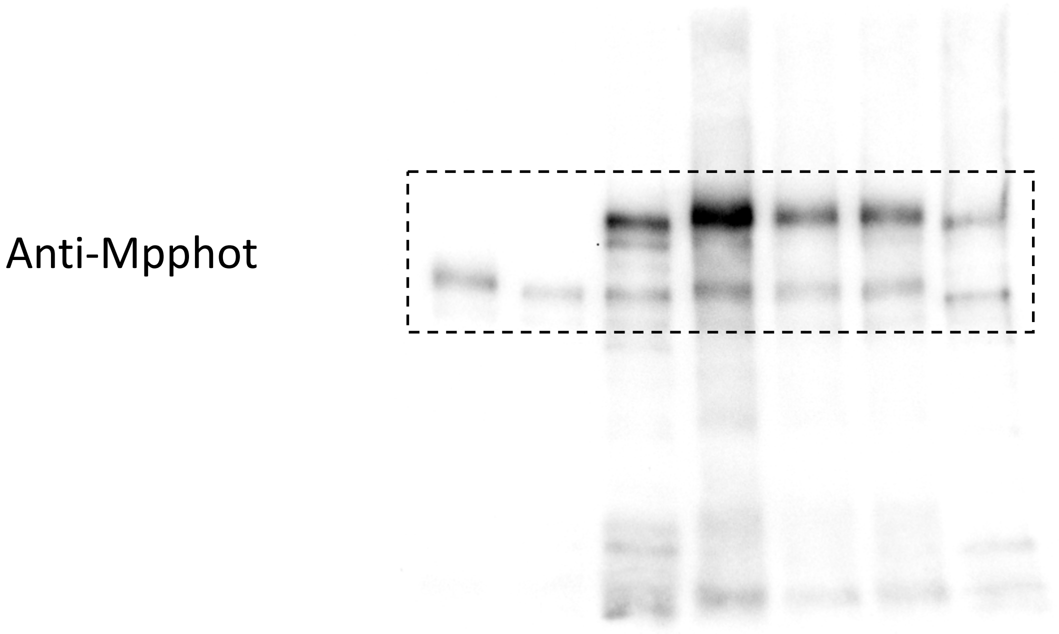

Figure 5A

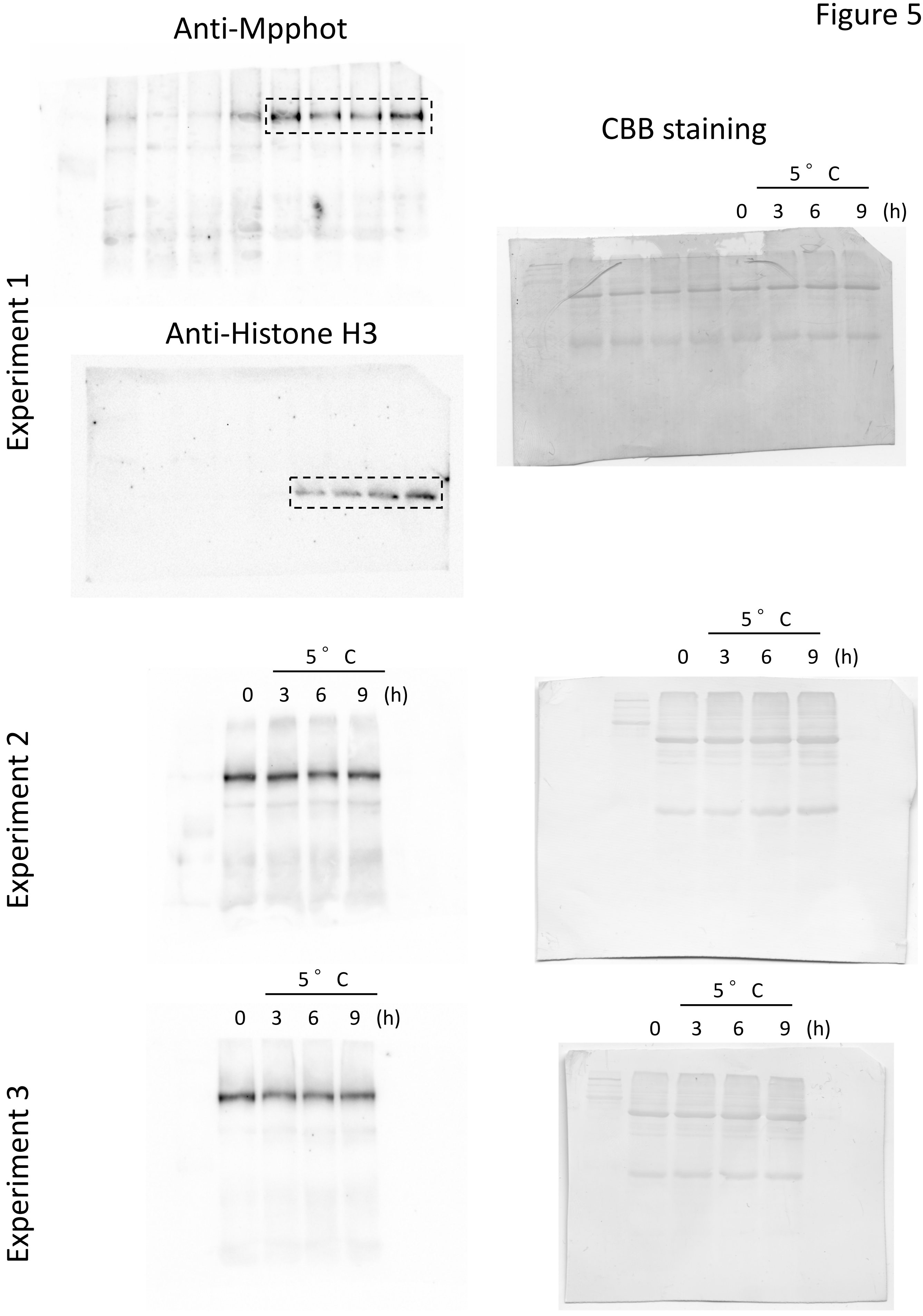

Anti-Mpphot
Anti-Histone H3
CBB staining
Experiment 1
Experiment 2
Experiment 3
5 ° C
0 3 6 9 (h)

Figure 5D

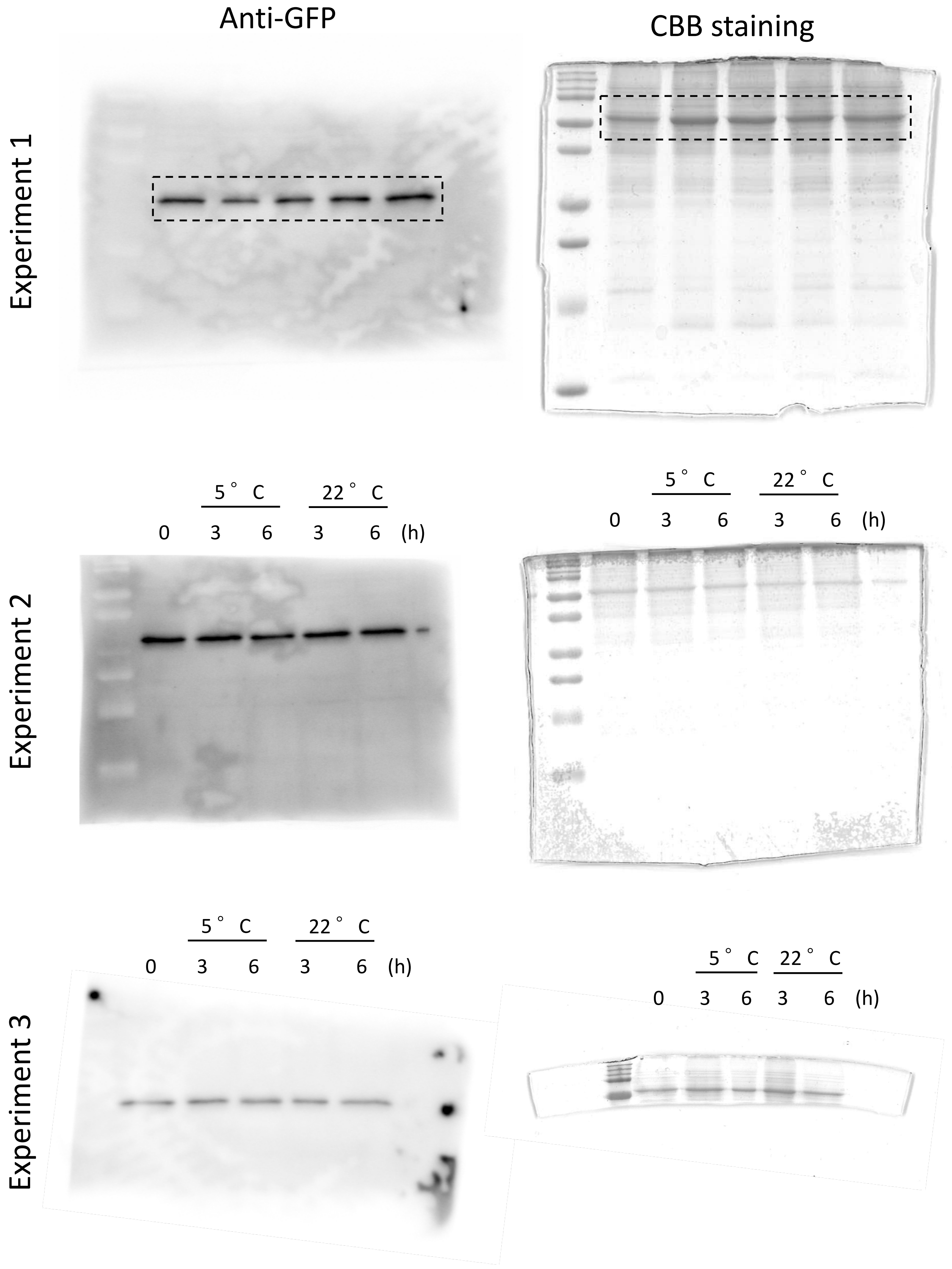
Anti-GFP
CBB staining
Experiment 1
Experiment 2
5 ° C
22 ° C
0
3
6
3
6
(h)
Experiment 3
5 ° C
22 ° C
0
3
6
3
6
(h)

Figure S1

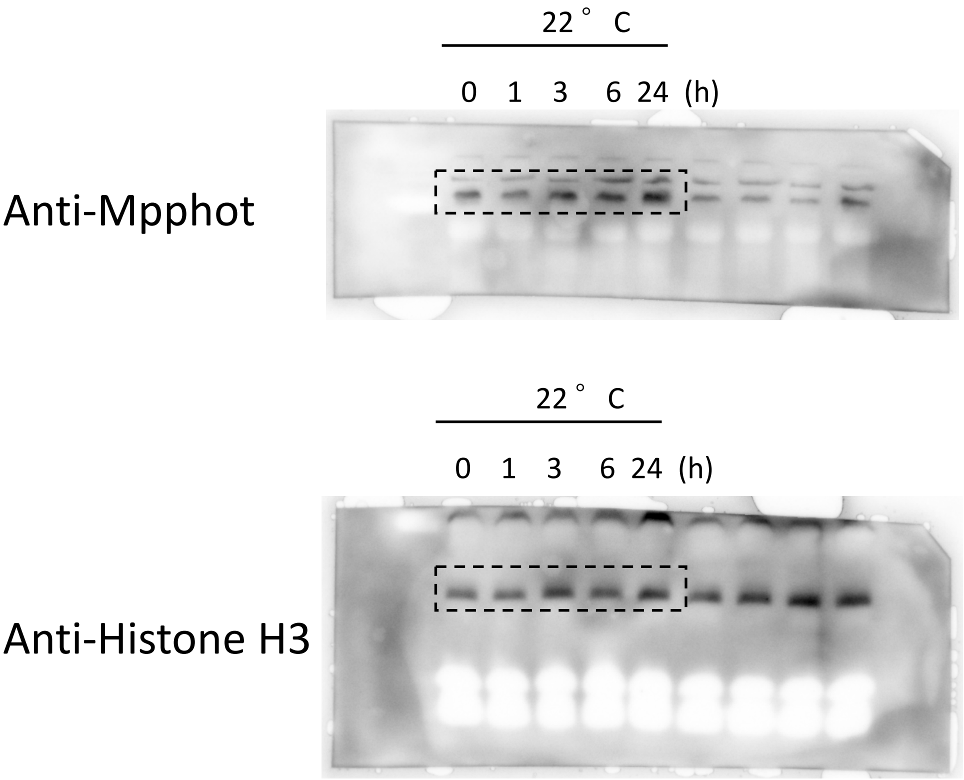

Supplement: S1 Raw images — (PDF) [file pone.0233302.s004.pdf]
